# Comparison of diabetes patients with “demyelinating” diabetic sensorimotor polyneuropathy to those diagnosed with CIDP

**DOI:** 10.1002/brb3.177

**Published:** 2013-09-22

**Authors:** Samantha K Dunnigan, Hamid Ebadi, Ari Breiner, Hans D Katzberg, Leif E Lovblom, Bruce A Perkins, Vera Bril

**Affiliations:** 1 Division of Neurology, Department of Medicine, University of TorontoToronto, Ontario, Canada; 2 Division of Endocrinology and Metabolism, Department of Medicine, University of TorontoToronto, Ontario, Canada

**Keywords:** CIDP, diabetic neuropathy, type 1 diabetes, type 2 diabetes

## Abstract

**Background:**

We have previously identified a subset of diabetic sensorimotor polyneuropathy (DSP) patients with probable demyelination related to poor glycemic control. We aimed to determine whether the clinical characteristics and electrodiagnostic classification of nerve injury in diabetes patients with “demyelinating” DSP (D-DSP) differed from those diagnosed with chronic inflammatory demyelinating polyneuropathy (CIDP) (CIDP + diabetes mellitus [DM]).

**Methods:**

D-DSP (56) and CIDP + DM (67) subjects underwent clinical examination and nerve conduction studies (NCS), and were compared using analysis of variance, contingency tables, and Kruskal–Wallis analyses.

**Results:**

Of the 123 subjects with a mean age of 60.5 ± 15.6 years and mean hemoglobin A_1c_ (HbA_1c_) of 8.2 ± 2.2%, 54% had CIDP + DM and 46% had D-DSP. CIDP + DM subjects were older (*P* = 0.0003), had shorter duration of diabetes (*P* = 0.005), and more severe neuropathy as indicated by Toronto Clinical Neuropathy Score (TCNS) (*P* = 0.003), deep tendon reflexes (*P* = 0.02), and vibration perception thresholds (VPT) (*P* = 0.01, *P* = 0.02). The mean HbA_1c_ value for D-DSP subjects (8.9 ± 2.3%) was higher than in CIDP + DM subjects (7.7 ± 2.0%, *P* = 0.02).

**Conclusions:**

The clinical phenotype and electrophysiological profile of CIDP + DM patients is marked by more severe neuropathy and better glycemic control than in patients with D-DSP. These findings indicate that these two conditions – despite similarities in their electrophysiological pattern of demyelination – likely differ in etiology.

## Introduction

Diabetic sensorimotor polyneuropathy (DSP) is a common complication of both type 1 and type 2 diabetes mellitus (DM) and is thought to occur due to hyperglycemia-related peripheral nerve damage. Classically, DSP results in axonal degeneration and progressive loss of nerve fibers, as indicated by reduced compound muscle action potential (CMAP) and sensory nerve action potential (SNAP) amplitudes, with normal or slightly reduced conduction velocities secondary to loss of the largest, fastest conducting axons (Behse et al. 1977; Dyck et al. 1986). For this reason, diabetes patients who have changes suggestive of demyelination on nerve conduction studies (NCS) are usually considered to have a superimposed immune-mediated polyneuropathy, such as chronic inflammatory demyelinating polyneuropathy (CIDP) (Van den Bergh et al. 2010). However, NCS changes suggestive of demyelination, such as conduction velocity slowing, have been demonstrated recently in patients with DSP and found to be related to glycemic control in those with type 1 diabetes (Dunnigan et al. 2013). Thus it becomes important to distinguish DSP from CIDP in diabetes patients as the latter may be amenable to treatment. Immunomodulatory therapies, including intravenous immunoglobulin (IVIg), corticosteroids, and plasma exchange can be effective treatments for CIDP in diabetes patients even in the presence of an underlying DSP (Van den Bergh et al. 2010; Latov 2011).

We sought to compare the clinical and electrodiagnostic features in patients with mild demyelinating changes in DSP (D-DSP) to those patients with diabetes diagnosed with CIDP (CIDP + DM). We aimed to determine if diabetes patients with D-DSP have unique profiles when compared to patients with CIDP + DM to allow the use of effective, targeted therapies.

## Materials and Methods

### Subjects

One-hundred and twenty-three diabetes subjects with polyneuropathy were accrued for this study in the neuromuscular clinic of Toronto General Hospital (TGH) at University Health Network (UHN). DSP subjects with type 1 (*n* = 27) and type 2 (*n* = 29) diabetes were seen between 2008 and 2012 as part of an ongoing longitudinal cohort study funded by the Juvenile Diabetes Research Foundation (Operating Grant No. 17-2008-715) and a cross-sectional cohort study funded by the Canadian Diabetes Association (Operating Grant No. OG-3-10-3123-BP). Diabetes subjects with CIDP were seen in clinic for management of their immune-mediated polyneuropathy between 1997 and 2012. All subjects were ≥18 years of age and had a confirmed diagnosis of type 1 or type 2 DM and either DSP or CIDP.

**Figure 1:**
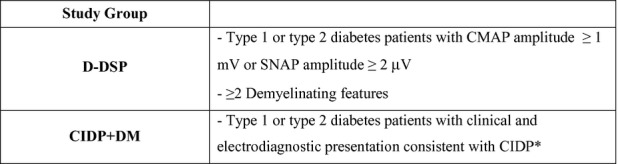
Schematic of two groups used to categorize patients as having demyelinating DSP (D-DSP) or diabetes and CIDP (CIDP + DM), based on a combination of amplitude, and latency and conduction velocity parameters. Demyelinating features are as follows: peroneal F-wave latency ≥61.6 msec; peroneal distal latency ≥6.7 msec; peroneal conduction velocity ≤37.5 m/sec; sural distal latency ≥3.7 msec; sural conduction velocity ≤36.0 m/sec. *As judged by a neuromuscular expert (VB) (Magda et al. 2003). CMAP, compound motor action potential; SNAP, sensory nerve action potential; DSP, diabetic sensorimotor polyneuropathy; CIDP, chronic inflammatory demyelinating polyneuropathy; DM, diabetes mellitus.

DM was diagnosed according to the American Diabetes Association criteria based on one of four abnormalities: hemoglobin A1c (HbA_1c_), fasting plasma glucose, random elevated glucose with symptoms, or abnormal oral glucose tolerance test (American Diabetes Association 2013). DSP was diagnosed according to the following criteria: at least one abnormal sural NCS result, one abnormal peroneal NCS result, and at least one neuropathic sign or symptom (England et al. 2005; Dyck et al. 2011). Criteria for mild D-DSP were defined previously (Dunnigan et al. 2013). In brief, we defined patients as having demyelination out of proportion to axonal loss (D-DSP) if amplitudes were preserved and at least two NCS parameters showed slowed conduction as suggested by the European Federation of Neurological Societies (EFNS) criteria for CIDP (Van den Bergh et al. 2010). CIDP was diagnosed in those patients having a clinical and electrodiagnostic presentation consistent with CIDP as judged by a neuromuscular expert (VB) (Magda et al. 2003). Criteria for the D-DSP and CIDP + DM study groups are shown in Figure 1.

As part of the initial cohort study, each participant underwent comprehensive medical and neurologic evaluation for the assessment of neuropathy-related symptoms and comorbidities, physical examination, and biochemical testing (HbA_1c_). Our current study involved the extraction of demographic data, clinical history, physical examination, laboratory results, and electrophysiologic data from the research database for DSP patients and previously coded charts for CIDP patients. None of the D-DSP subjects had a diagnosis of immune-mediated polyneuropathy or CIDP. The CIDP + DM group lacked specific tests or biomarkers to confirm the diagnosis of CIDP other than NCS and expert opinion. The Research Ethics Board of the University Health Network approved the current study protocol.

Subjects were evaluated for neuropathy by neurological examination, the 19-point Toronto Clinical Neuropathy Score (TCNS), vibration perception thresholds (VPT), and sural and peroneal NCS (Bril and Perkins 2002). We restricted this comparison to lower limb NCS parameters as the battery of NCS testing differed between D-DSP and CIDP + DM in our patient population. NCS were performed using the Sierra Wave instrument (Cadwell Laboratories Inc., Kennewick, WA). Age- and height-adjusted NCS reference values were used, according to the standards of the TGH (UHN) electrophysiology laboratory. Limb temperature was measured prior to NCS, and if required, warming was performed to ensure a surface temperature of ≥32.0°C in the hands and ≥31.0°C in the feet.

### Statistical analysis

Statistical analysis was performed using JMP (version 9.0.2 for Macintosh, from SAS, SAS Institute Inc., Toronto, Canada). Demographic data were expressed as means ± standard deviation (SD) for normally distributed data, or median and interquartile range [IQR] for data not normally distributed. Differences in categorical variables were assessed using the *χ*^2^-test, while differences in continuous variables were assessed using the analysis of variance (ANOVA) or the Kruskal–Wallis test for nonparametric data. Correlation between peroneal compound motor action potential (CMAP) amplitude and conduction velocity was investigated using linear regression methods. *P*-values less than 0.05 were considered significant.

## Results

The demographic data of the 123 type 1 and type 2 diabetes subjects categorized as having D-DSP or CIDP + DM are shown in Table 1. The 123 subjects had a mean age of 60.5 ± 15.6 years and mean HbA_1c_ of 8.2 ± 2.2% (66 ± 24 mmol/mol). Of these subjects, 67 (54%) had CIDP + DM and 56 (46%) had D-DSP. CIDP + DM subjects were older (*P* = 0.0003) and had shorter duration of diabetes (*P* = 0.005) and higher diastolic blood pressures (*P* = 0.04) than D-DSP subjects. Subjects did not differ in terms of body mass index (BMI) (*P* = 0.51), systolic blood pressures (*P* = 0.91), and upper limb VPT (*P* = 0.11, *P* = 0.13), or in the presence of retinopathy (*P* = 0.24), nephropathy (*P* = 0.70), or hypertension (*P* = 0.11).

**Table 1:** Clinical and electrodiagnostic features of 67 CIDP + DM and 56 type 1 and type 2 diabetes D-DSP subjects according to study criteria for demyelinating neuropathy

	CIDP + DM and type 1 and type 2 diabetes D-DSP subjects (*n* = 123)		
		
	CIDP + DM	D-DSP	ANOVA *P*-value for trend
*n*	67	56	
Age (years)^1^	65.1 ± 13.7	55.0 ± 16.0	0.0003
Male sex, *n* (%)	46 (69%)	37 (67%)	0.87
BMI (kg/m^2^)	27.7 ± 6.0	28.9 ± 5.6	0.51
Type 2 DM, *n* (%)	65 (97%)	29 (52%)	<0.0001
Duration DM (years)	16.5 ± 13.5	24.0 ± 15.6	0.005
Duration PNP (years)	9.93 ± 8.5	7.64 ± 5.6	0.20
Systolic blood pressure (mmHg)	140.8 ± 21.8	140.3 ± 18.0	0.91
Diastolic blood pressure (mmHg)	81.5 ± 12.8	76.4 ± 9.9	0.04
VPT upper right	7.61 ± 4.6	6.29 ± 4.1	0.11
VPT upper left	7.63 ± 5.2	6.30 ± 4.1	0.13
VPT lower right	31.4 ± 13.4	25.3 ± 13.1	0.01
VPT lower left	30.2 ± 12.9	24.7 ± 12.4	0.02
TCNS, median [IQR]	13 [9, 16]	11 [7, 14]	0.003
Retinopathy, *n* (%)	11 (16%)	14 (25%)	0.24
Nephropathy, *n* (%)	8 (12%)	8 (14%)	0.70
Hypertension, *n* (%)	42 (63%)	27 (48%)	0.11
HbA_1c_, % (mmol/mol)^2^	7.7 ± 2.0 (61 ± 21.9)	8.9 ± 2.3 (74 ± 25.1)	0.02
Nerve conduction parameters			
Sural nerve amplitude potential (μV)	2.40 ± 3.0	2.29 ± 1.8	0.82
Sural nerve distal latency (msec)	3.59 ± 0.6	3.72 ± 0.4	0.23
Sural nerve conduction velocity (m/sec)	38.6 ± 5.4	37.9 ± 3.6	0.50
Peroneal nerve amplitude potential (mV) – ankle	1.97 ± 2.4	2.15 ± 1.5	0.63
Peroneal nerve amplitude potential (mV) – knee	1.84 ± 2.4	1.84 ± 1.3	0.98
Peroneal nerve distal latency (msec)	5.97 ± 1.4	5.22 ± 1.0	0.002
Peroneal nerve conduction velocity (m/sec)	32.4 ± 6.4	35.2 ± 3.4	0.006
Peroneal nerve F-wave (msec)	59.2 ± 16.1	62.5 ± 4.9	0.38
Conduction block (%)^3^	9.8 ± 44.1	14.2 ± 14.0	0.49

Data are means ± SD unless otherwise indicated. Differences in categorical variables were assessed in three-group comparisons using the *χ*^2^-test, while differences in continuous variables were assessed using the ANOVA except in the case of TCNS in which the Kruskal–Wallis test was applied. Toronto Clinical Neuropathy Score (TCNS) is a clinical indicator of the severity of neuropathy, with 0–4, 5–8, and ≥9 indicating no, mild, and moderate to severe neuropathy. Values less than 5 are normal. BMI, body mass index; DM, diabetes mellitus; PNP, polyneuropathy; VPT, vibration perception threshold; DSP, diabetic sensorimotor polyneuropathy; CIDP, chronic inflammatory demyelinating polyneuropathy; ANOVA, analysis of variance; IQR, interquartile range.

1 The mean age for the 123 CIDP and DSP subjects was 60.5 ± 15.6 years.

2 The mean HbA_1c_, indicating the percentage of hemoglobin A_1c_, for 82 of the 123 CIDP and DSP subjects was 8.2 ± 2.2% (66 ± 24 mmol/mol).

3 Conduction block (%) is based on the ratio of the [distal – proximal peroneal nerve amplitude]/distal peroneal nerve amplitude × 100.

The severity of neuropathy was increased in CIDP + DM subjects as indicated by the higher TCNS (13 [9, 16], 11 [7, 14], *P* = 0.003), greater impairment of lower limb reflexes (*P* = 0.02) and more elevated lower limb VPT (*P* = 0.01, *P* = 0.02). A detailed comparison of lower limb reflexes is shown in Table 2. A higher percentage of patients with CIDP + DM had loss of reflexes at knees and ankles compared to D-DSP. Despite 36% of D-DSP subjects reporting a complaint of weakness on TCNS, these subjects were free of objective weakness on clinical examination. In the CIDP + DM group, 84% reported a complaint of weakness on TCNS and 63% had objective weakness on clinical examination. Of the CIDP + DM patients who had objective weakness on clinical examination, the mean for proximal versus distal muscle groups of the upper limb was 4.77 ± 0.4 versus 4.19 ± 0.7, and the mean grade for proximal versus distal muscle groups of the lower limb was 4.46 ± 0.8 versus 4.24 ± 1.1, where 5 indicates normal strength.

**Table 2:** Lower limb reflexes on TCNS of 121 CIDP + DM and type 1 and type 2 diabetes D-DSP subjects

	Reflexes on TCNS											
	
	Knee jerk – right (%)			Knee jerk – left (%)			Ankle jerk – right (%)			Ankle jerk – left (%)		
				
	N^1^	S^2^	A^3^	N	S	A	N	S	A	N	S	A
Study groups (*n*)												
CIDP + DM (67)	35.8	29.8	34.3	34.3	28.4	37.3	14.9	13.4	71.7	15.2	13.6	71.2
D-DSP (54)	50.0	35.2	14.8	48.1	35.2	16.7	13.0	40.7	46.3	14.8	35.2	50.0
*P*-value	0.04			0.04			0.002			0.02		

TCNS, Toronto Clinical Neuropathy Score; DM, diabetes mellitus; DSP, diabetic sensorimotor polyneuropathy; CIDP, chronic inflammatory demyelinating polyneuropathy.

1 Normal reflexes.

2 Sluggish reflexes.

3 Absent reflexes.

CIDP + DM subjects had increased peroneal distal motor latencies (5.97 ± 1.4, 5.22 ± 1.0, *P* = 0.002) and slower peroneal motor conduction velocities (32.4 ± 6.4, 35.2 ± 3.4, *P* = 0.006) than D-DSP subjects. However, the distal peroneal CMAP amplitude (*P* = 0.63) and F-wave latencies (*P* = 0.38), as well as sural SNAP amplitudes (*P* = 0.82), distal sensory latencies (*P* = 0.23), and sensory conduction velocities (*P* = 0.50) showed no difference. No difference in conduction block was observed between study groups. A positive linear correlation between peroneal distal CMAP amplitude and conduction velocity was found among D-DSP and CIDP + DM subgroups (*P* = 0.017, *P* = 0.03), with similar weak correlation strengths for D-DSP (*r*^2^ = 0.09) and CIDP + DM (*r*^2^ = 0.1) patients. Most importantly, the mean HbA_1c_ value for D-DSP subjects (8.9 ± 2.3% [74 ± 25.1 mmol/mol]) was significantly higher than CIDP + DM subjects (7.7 ± 2.0% [61 ± 21.9 mmol/mol], *P* = 0.02).

When the analyses were repeated for the CIDP + DM subjects compared to type 1 (Table 3) and type 2 (Table 4) D-DSP subjects separately, similar findings were demonstrated with the exception that the differences in HbA_1c_ values were found only between CIDP + DM patients and type 1 D-DSP subjects (7.7 ± 2.0 [61 ± 21.9 mmol/mol], 9.6 ± 2.4 [81 ± 26.2 mmol/mol], *P* = 0.003) (Table 3). Type 1 diabetes D-DSP patients also had a higher occurrence of retinopathy (*P* = 0.04) and a lower occurrence of hypertension (*P* = 0.02) than CIDP + DM patients.

**Table 3:** Clinical and electrodiagnostic features of 67 CIDP + DM and 27 type 1 diabetes D-DSP subjects according to study criteria for demyelinating neuropathy

	CIDP + DM and type 1 diabetes D-DSP subjects (*n* = 94)		
		
	CIDP + DM	D-DSP	ANOVA *P*-value for trend
*n*	67	27	
Age (years)^1^	65.1 ± 13.7	48.0 ± 17.2	<0.0001
Male sex, *n* (%)	46 (69%)	13 (48%)	0.87
BMI (kg/m^2^)	27.7 ± 6.0	26.6 ± 5.6	0.58
Type 2 DM, *n* (%)	65 (97%)	0 (0%)	<0.0001
Duration DM (years)	16.5 ± 13.5	33.1 ± 14.8	<0.0001
Duration PNP (years)	9.93 ± 8.5	9.93 ± 5.5	0.99
Systolic blood pressure (mmHg)	140.8 ± 21.8	140.0 ± 18.5	0.88
Diastolic blood pressure (mmHg)	81.5 ± 12.8	75.9 ± 9.6	0.06
VPT upper right	7.61 ± 4.6	6.63 ± 5.3	0.38
VPT upper left	7.63 ± 5.2	6.60 ± 5.2	0.40
VPT lower right	31.4 ± 13.4	21.5 ± 11.1	0.001
VPT lower left	30.2 ± 12.9	20.8 ± 10.6	0.001
TCNS, median [IQR]	13 [9, 16]	11 [6, 15]	0.01
Retinopathy, *n* (%)	11 (16%)	10 (37%)	0.04
Nephropathy, *n* (%)	8 (12%)	3 (11%)	0.91
Hypertension, *n* (%)	42 (63%)	10 (37%)	0.02
HbA_1c_, % (mmol/mol)^2^	7.7 ± 2.0 (61 ± 21.9)	9.6 ± 2.4 (81 ± 26.2)	0.003
Nerve conduction parameters			
Sural nerve amplitude potential (μV)	2.40 ± 3.0	2.43 ± 2.0	0.96
Sural nerve distal latency (msec)	3.59 ± 0.6	3.68 ± 0.3	0.52
Sural nerve conduction velocity (m/sec)	38.6 ± 5.4	38.2 ± 3.1	0.82
Peroneal nerve amplitude potential (mV) – ankle	1.97 ± 2.4	1.96 ± 1.4	0.99
Peroneal nerve amplitude potential (mV) – knee	1.84 ± 2.4	1.66 ± 1.3	0.71
Peroneal nerve distal latency (msec)	5.97 ± 1.4	5.45 ± 1.2	0.12
Peroneal nerve conduction velocity (m/sec)	32.4 ± 6.4	34.8 ± 4.3	0.084
Peroneal nerve F-wave (msec)	59.2 ± 16.1	62.7 ± 7.4	0.61
Conduction block (%)^3^	9.77 ± 44.1	17.7 ± 14.0	0.39

Data are means ± SD unless otherwise indicated. Differences in categorical variables were assessed in three-group comparisons using the *χ*^2^-test, while differences in continuous variables were assessed using the ANOVA except in the case of TCNS in which the Kruskal–Wallis test was applied. Toronto Clinical Neuropathy Score (TCNS) is a clinical indicator of the severity of neuropathy, with 0–4, 5–8, and ≥9 indicating no, mild, and moderate to severe neuropathy. Values less than 5 are normal. BMI, body mass index; DM, diabetes mellitus; PNP, polyneuropathy; VPT, vibration perception threshold; DSP, diabetic sensorimotor polyneuropathy; CIDP, chronic inflammatory demyelinating polyneuropathy; ANOVA, analysis of variance; IQR, interquartile range.

1 The mean age for the 94 CIDP and DSP subjects was 60.2 ± 16.6 years.

2 The mean HbA_1c_, indicating the percentage of hemoglobin A_1c_, for 67 of the 94 CIDP and DSP subjects was 8.2 ± 2.3% (66 ± 25.1 mmol/mol).

3 Conduction block (%) is based on the ratio of the [distal – proximal peroneal nerve amplitude]/distal peroneal nerve amplitude × 100.

**Table 4:** Clinical and electrodiagnostic features of 67 CIDP + DM and 29 type 2 diabetes D-DSP subjects according to study criteria for demyelinating neuropathy

	CIDP + DM and type 2 diabetes D-DSP subjects (*n* = 96)		
		
	CIDP + DM	D-DSP	ANOVA *P*-value for trend
*n*	67	29	
Age (years)^1^	65.1 ± 13.7	61.5 ± 11.8	0.22
Male sex, *n* (%)	46 (69%)	24 (86%)	0.073
BMI (kg/m^2^)	27.7 ± 6.0	31.5 ± 4.4	0.043
Type 2 DM, *n* (%)	65 (97%)	29 (100%)	0.23
Duration DM (years)	16.5 ± 13.5	15.2 ± 10.6	0.66
Duration PNP (years)	9.93 ± 8.5	5.36 ± 4.8	0.057
Systolic blood pressure (mmHg)	140.8 ± 21.8	140.8 ± 17.7	0.21
Diastolic blood pressure (mmHg)	81.5 ± 12.8	77.1 ± 10.5	0.21
VPT upper right	7.61 ± 4.6	5.94 ± 2.5	0.082
VPT upper left	7.63 ± 5.2	5.99 ± 2.5	0.12
VPT lower right	31.4 ± 13.4	29.1 ± 14.0	0.46
VPT lower left	30.2 ± 12.9	28.5 ± 13.0	0.58
TCNS, median [IQR]	13 [9, 16]	11 [8, 14]	0.039
Retinopathy, *n* (%)	11 (16%)	4 (14%)	0.74
Nephropathy, *n* (%)	8 (12%)	5 (17%)	0.49
Hypertension, *n* (%)	42 (63%)	17 (57%)	0.71
HbA_1c_, % (mmol/mol)^2^	7.7 ± 2.0 (61 ± 21.9)	8.1 ± 2.0 (65 ± 21.9	0.45
Nerve conduction parameters			
Sural nerve amplitude potential (μV)	2.40 ± 3.0	2.18 ± 1.6	0.71
Sural nerve distal latency (msec)	3.59 ± 0.6	3.75 ± 0.4	0.25
Sural nerve conduction velocity (m/sec)	38.6 ± 5.4	37.6 ± 4.0	0.45
Peroneal nerve amplitude potential (mV) – ankle	1.97 ± 2.4	2.31 ± 1.6	0.47
Peroneal nerve amplitude potential (mV) – knee	1.84 ± 2.4	2.0 ± 1.3	0.75
Peroneal nerve distal latency (msec)	5.97 ± 1.4	5.01 ± 0.67	0.001
Peroneal nerve conduction velocity (m/sec)	32.4 ± 6.4	35.5 ± 2.5	0.017
Peroneal nerve F-wave (msec)	59.2 ± 16.1	62.4 ± 3.7	0.47
Conduction block (%)^3^	9.77 ± 44.1	10.9 ± 13.5	0.89

Data are means ± SD unless otherwise indicated. Differences in categorical variables were assessed in three-group comparisons using the *χ*^2^-test, while differences in continuous variables were assessed using the ANOVA except in the case of TCNS in which the Kruskal–Wallis test was applied. Toronto Clinical Neuropathy Score (TCNS) is a clinical indicator of the severity of neuropathy, with 0–4, 5–8, and ≥9 indicating no, mild, and moderate to severe neuropathy. Values less than 5 are normal. BMI, body mass index; DM, diabetes mellitus; PNP, polyneuropathy; VPT, vibration perception threshold; DSP, diabetic sensorimotor polyneuropathy; CIDP, chronic inflammatory demyelinating polyneuropathy; ANOVA, analysis of variance; IQR, interquartile range.

1 The mean age for the 96 CIDP and DSP subjects was 64.0 ± 13.2 years.

2 The mean HbA_1c_, indicating the percentage of hemoglobin A_1c_, for 65 of the 96 CIDP and DSP subjects was 7.8 ± 2.0% (62 ± 21.9 mmol/mol).

3 Conduction block (%) is based on the ratio of the [distal – proximal peroneal nerve amplitude]/distal peroneal nerve amplitude × 100.

## Discussion

We examined a cohort of type 1 and type 2 diabetes patients with D-DSP or CIDP + DM to compare their clinical characteristics and electrodiagnostic classification of nerve injury and observed that D-DSP patients have a unique clinical profile when compared to patients with CIDP + DM. Specifically, CIDP + DM patients are older, have better glycemic control, a shorter duration of diabetes, and more severe nerve injury than patients with D-DSP.

In a previous study, different electrophysiologic behaviors were found to be linked to metabolic control in D-DSP such that demyelinating change on NCS indicates worse control and may afford the opportunity for intervention (Dunnigan et al. 2013). In this study, CIDP + DM patients had even greater degrees of demyelination but better glycemic control, indicating that different pathophysiological mechanisms may account for demyelinating features in these disorders. In contrast to CIDP + DM, the D-DSP group had higher HbA_1c_ values and lacked weakness on examination, so the demyelinating features on NCS in these patients are more likely to be caused by metabolic rather than inflammatory nerve damage. The higher HbA_1c_ values in D-DSP patients suggests that suboptimal glycemic control plays a prominent role in the observed conduction slowing compared to CIDP + DM patients who likely have other factors leading to conduction slowing.

Existing criteria for the diagnosis of CIDP are highly specific (Rajabally et al. 2009) and may lack the necessary sensitivity to diagnose a separate demyelinating neuropathy in DSP patients and so the diagnosis of CIDP + DM is difficult because of overlap in clinical and electrophysiological characteristics in these neuropathies. Previous nerve fiber injury due to diabetes may mask novel demyelinating changes related to immune-mediated nerve injury. Thus, it is probable that highly specific criteria for CIDP in DSP patients will have very low sensitivity. We have observed in diabetes patients, electrophysiological and clinical findings atypical for classic DSP although insufficient for existing CIDP criteria. For example, we observed a reduction in conduction velocity in DSP out of proportion to the axonal loss, but still not in the range of defined criteria for CIDP. That raised the possibility of an unexpected degree of demyelination in the context of DSP, and we discovered that this group of patients had type 1 diabetes and suboptimal glycemic control. These findings could indicate abnormal immune mechanisms in type 1 diabetes patients producing both findings, or relate to more sensitivity to metabolic damage of the Schwann cells in type 1 diabetes patients. Our current findings show even greater degrees of demyelination in the CIDP + DM group that are associated with a more severe neuropathy phenotype (greater weakness, more abnormal reflexes, higher TCNS scores, and more abnormal NCS), but less impaired glycemic control, supporting the diagnosis of an immune-mediated polyneuropathy rather than DSP.

Limitations of the current study are as follows:

1. Referral bias – CIDP + DM patients were accrued differently than D-DSP as they were referred based on the clinical suspicion of CIDP and may have a greater severity of disease. Also, given the difference in accrual intervals of about 10 years, bias regarding improved management may exist.
2. NCS do not necessarily define “demyelination” – rather, they may indicate myelin or nodal dysfunction. Although the NCS patterns are similar between the two conditions, there may be structural differences that could be discerned by other tests such as ultrasound, biopsy, or magnetic resonance imaging (MRI). Also, as clinicians might use NCS in the upper extremities to distinguish CIDP from D-DSP, exclusion of upper limb NCS may limit the observations.
3. Misclassification is a potential error – there are no biomarkers to make a definitive diagnosis of CIDP and demyelination or conduction slowing on NCS is not a specific finding. However, the differences in clinical phenotype observed between the groups support the diagnostic classification. Also, the degree of demyelination used to define CIDP in this study are not as strict as in published criteria, but existing criteria are accepted as lacking high sensitivity and recent approaches employ more relaxed criteria (Koski et al. 2009; Brannagan 2011). In addition, although not all of our patients in the CIDP + DM group had weakness on examination, sensory variants of CIDP are well recognized, lending support to inclusion of these patients in this group.
4. The treatment of the TCNS as a linear scale despite that it is a composite multiitem measure and the assumption that the response options per item have equal numerical value may not be valid (Stucki et al. 1996).

The differentiation of CIDP from DSP in patients with diabetes is important due to the implications for therapy and prognosis. This study helps to define the clinical phenotype and electrophysiological profile of CIDP + DM patients and shows that they differ in DSP patients, even those with D-DSP. Future work directed toward finding a definitive biomarker for the diagnosis of immune-mediated polyneuropathies would have major therapeutic implications for all patients.
